# Trauma and sexual risk behaviors in an adolescent victim of sexual abuse: A case report

**DOI:** 10.1192/j.eurpsy.2021.1998

**Published:** 2021-08-13

**Authors:** L. Borges

**Affiliations:** Psiquiatria, CHUA - Unidade de Portimão, Portimão, Portugal

**Keywords:** post-traumatic stress disorder, sexual abuse, sexual risk behaviors, trauma

## Abstract

**Introduction:**

Childhood and adolescence sexual abuse (CSA) is a risk factor for psychological trauma and a strong predictor of lifetime psychopathology, including depression, anxiety, inappropriate sexual behavior, anger, guilt, shame and other emotional and relationship problems.

**Objectives:**

Describe a clinical case of a sexually abused adolescent admitted in a psychiatric unit for young adults and to correlate sexual abuse with trauma and sexual risk behaviors.

**Methods:**

The data was collected through clinical and family interviews. The revision was made with the search terms “trauma”, “child and adolescence sexual abuse”, “sexual risk behaviors” in scientific databases.

**Results:**

16 year-old girl, high-school student, living with her nuclear family, was admitted in a psychiatric hospital with feelings of sadness and anxiety since the previous month, that lead to a voluntary medicine ingestion. She has been continuously sexually abused from the age of 12 to 16 by an older man, and once by her cousin and his friends. Since than, she refers feelings of anger, sadness, dissociative symptoms and intrusive images and nightmares related to the abuses, and continues to seek attention from older men. With medication and individual and family psychotherapeutic interventions, depressive, anxiety and dissociative symptoms have improved.

**Conclusions:**

Literature concludes that there’s a strong correlation between CSA, trauma and sexual risk behaviors throughout adulthood. In fact, our patient met criteria for Pos-traumatic Stress Disorder and has sexual risk behaviors that must be worked through therapy. Due to it’s complexity, treatment of the adolescent and familial system after sexual abuse is multifaceted and requires a biopsychosocial approach.

**Disclosure:**

No significant relationships.

